# Supplementary material for: Effective simulations of interacting active droplets
Source: Sci Rep. 2023 Jan 13;13:733. doi: 10.1038/s41598-023-27630-3 (PMC9839783; doi:10.1038/s41598-023-27630-3)
Supplement: Supplementary file 1 — Supplementary Information. [file 41598_2023_27630_MOESM1_ESM.pdf]

Supplementary Information for "Effective simulations of interacting active droplets"

Ajinkya Kulkarni,[1] Estefania Vidal-Henriquez,[1] and David Zwicker[1]

[1] *Max Planck Institute for Dynamics and Self-Organization, Am Fassberg 17, 37077 Göttingen, Germany*

(Dated: November 28, 2022)

I. INTERFACIAL VELOCITY OF DROPLETS

We investigate the dynamics of the droplet interface by analyzing an infinitesimal cuboid placed on the interface. The cuboid is aligned with the interface and covers an area element $\mathrm{d}A$ of it. It protrudes by distances ϵ_{in} and ϵ_{out} inside and outside the interface, respectively, so its volume is given by $(\epsilon_{\mathrm{in}}+\epsilon_{\mathrm{out}})\,\mathrm{d}A$. Keeping the cuboid fixed in space, the distances change by the interfacial speed v_{n} in the normal direction, $\partial_t\epsilon_{\mathrm{in}} = -\partial_t\epsilon_{\mathrm{out}} = v_{\mathrm{n}}$. We use this to express the time derivative of the amount of material in the cuboid, $\Phi = (\epsilon_{\mathrm{in}}\,\phi^{\mathrm{eq}}_{\mathrm{in}} + \epsilon_{\mathrm{out}}\,\phi^{\mathrm{eq}}_{\mathrm{out}})\,\mathrm{d}A$, as $\partial_t\Phi = \left(\phi^{\mathrm{eq}}_{\mathrm{in}}v_{\mathrm{n}} + \epsilon_{\mathrm{in}}\partial_t\phi^{\mathrm{eq}}_{\mathrm{in}} - \phi^{\mathrm{eq}}_{\mathrm{out}}v_{\mathrm{n}} + \epsilon_{\mathrm{out}}\partial_t\phi^{\mathrm{eq}}_{\mathrm{out}}\right)\mathrm{d}A$. On the other hand, the amount Φ changes due to material fluxes, $\partial_t\Phi \approx (\boldsymbol{j}_{\mathrm{in}} - \boldsymbol{j}_{\mathrm{out}})\cdot\boldsymbol{n}\,\mathrm{d}A$, where we neglect chemical reactions in the infinitesimal volume element and divergences of fluxes tangential to the interface. Equating the two expressions for $\partial_t\Phi$ in the limit of small ϵ_{in} and ϵ_{out}, we arrive at Eq. (8) of the main text.

II. GROWTH AND DRIFT OF DROPLETS

We here derive the expressions for the growth speed and drift rate of droplets of arbitrary shape. For simplicity, we focus on $d = 3$ dimensions, but the derivation works analogously in all dimensions. The 2D surface of a 3D droplet can be parameterized by points $\boldsymbol{R}(\theta,\varphi)$ with parameters θ and φ. For simplicity, we consider convex droplet shapes, so we can place a spherical coordinate system inside the droplet and write $\boldsymbol{R}(\theta,\varphi,t) = P(\theta,\varphi,t)\,\boldsymbol{e}_r$, where θ and φ denote the typical angles, $\boldsymbol{e}_r$ is the radial unit vector of the droplet surface, and $P(\theta,\varphi,t)$ denotes the distance of the surface from the origin. The droplet volume reads $V = d^{-1}\int P^{\,d}\,\mathrm{d}\Omega$, where $\mathrm{d}\Omega$ is the solid angle element, which reads $\mathrm{d}\Omega = \sin\theta\,\mathrm{d}\theta\mathrm{d}\varphi$ for spherical coordinates. The volume changes in time as $\partial_t V = \int(\partial_t P)\,P^{\,d-1}\,\mathrm{d}\Omega$, where $\partial_t P$ is the interfacial speed in the radial direction. We obtain it from the normal speed v_{n}, given by Eq. (8) in the main text, using the geometric relation $v_{\mathrm{n}} = (\partial_t P)\,\boldsymbol{e}_r\cdot\boldsymbol{n}$, which implies $\partial_t P = v_{\mathrm{n}}/(\boldsymbol{e}_r\cdot\boldsymbol{n})$. Additionally, the differential element $\mathrm{d}\Omega$ can be linked to the surface area element $\mathrm{d}A$ by $P^{\,d-1}\mathrm{d}\Omega = \boldsymbol{e}_r\cdot\boldsymbol{n}\,\mathrm{d}A$. Taken together, the rate of change of the radius R of a sphere with volume V, given by $\partial_t R = (\partial_t V)/S$ for the surface area S, can then be expressed as Eq. (9a) in the main text.

Similarly, we analyze the center-of-mass position $\boldsymbol{x} = [(d{+}1)V]^{-1}\int P^{\,d+1}\boldsymbol{e}_r\,\mathrm{d}\Omega$. We find $\partial_t(\boldsymbol{x}V) = \int P^{\,d}\,(\partial_t P)\,\boldsymbol{e}_r\,\mathrm{d}\Omega = \int P v_{\mathrm{n}}\boldsymbol{e}_r\,\mathrm{d}A$. If the droplet is initially centered on the origin, $\boldsymbol{x} = 0$, this implies $\partial_t\boldsymbol{x} = V^{-1}\int P v_{\mathrm{n}}\boldsymbol{e}_r\,\mathrm{d}A$. This expression is equivalent to Eq. (9b) of the main text for spherical droplets, where $P(\theta,\varphi) = R$, $\boldsymbol{e}_r = \boldsymbol{n}$, and $R/V = d/S$.

III. FLUXES INSIDE THE DROPLET

We determine the flux inside the droplet from the stationary form of the volume fraction field ϕ_{in} inside the droplet. We thus solve for the stationary state of Eq. (5) in the main text in a coordinate system with angular symmetry centered at the droplet, so that fields only depend on the radial coordinate r. Considering the boundary conditions $\phi_{\mathrm{in}}(R) = \phi^{\mathrm{eq}}_{\mathrm{in}}$ and $\partial_r\phi_{\mathrm{in}}(0) = 0$, as well as $R \ll \xi_{\mathrm{in}} = \sqrt{D_{\mathrm{in}}/|k_{\mathrm{in}}|}$, we find for 1, 2 and 3 dimensions

$$\phi_{\mathrm{in,1D}}(r) = \phi^0_{\mathrm{in}} + \frac{s(\phi^0_{\mathrm{in}})}{k_{\mathrm{in}}} - \frac{s(\phi^{\mathrm{eq}}_{\mathrm{in}})}{k_{\mathrm{in}}}\frac{\cosh\left(\frac{r}{\xi_{\mathrm{in}}}\right)}{\cosh\left(\frac{R}{\xi_{\mathrm{in}}}\right)} \tag{1a}$$

$$\phi_{\mathrm{in,2D}}(r) = \phi^0_{\mathrm{in}} + \frac{s(\phi^0_{\mathrm{in}})}{k_{\mathrm{in}}} - \frac{s(\phi^{\mathrm{eq}}_{\mathrm{in}})}{k_{\mathrm{in}}}\frac{I_0\left(\frac{r}{\xi_{\mathrm{in}}}\right)}{I_0\left(\frac{R}{\xi_{\mathrm{in}}}\right)} \tag{1b}$$

$$\phi_{\mathrm{in,3D}}(r) = \phi^0_{\mathrm{in}} + \frac{s(\phi^0_{\mathrm{in}})}{k_{\mathrm{in}}} - \frac{R}{r}\frac{s(\phi^{\mathrm{eq}}_{\mathrm{in}})}{k_{\mathrm{in}}}\frac{\sinh\left(\frac{r}{\xi_{\mathrm{in}}}\right)}{\sinh\left(\frac{R}{\xi_{\mathrm{in}}}\right)}\,, \tag{1c}$$

where I_0 is the modified Bessel function of the first kind. In all dimensions, the fluxes inside the interface are approximately $\boldsymbol{j}_{\mathrm{in}} \approx [-D_{\mathrm{in}}\boldsymbol{\nabla}\phi_{\mathrm{in}}(R)]\,\boldsymbol{n} \approx \frac{R}{d}\,\left[s(\phi_{\mathrm{in}}^0) - k_{\mathrm{in}}(\phi_{\mathrm{in}}^{\mathrm{eq}} - \phi_{\mathrm{in}}^0)\right]\,\boldsymbol{n}$, where d is the space dimension.

IV. FLUXES IN DROPLET SHELL SECTORS

We consider a droplet of radius R with a shell of thickness ℓ. To determine the profile $\phi_{\mathrm{out}}^{(m)}(r)$ in the m-th shell sector, we solve Eq. (7) in the main text in stationary state. We assume spherical symmetry and impose the boundary conditions $\phi_{\mathrm{out}}^{(m)}(R) = \phi_{\mathrm{out}}^{\mathrm{eq}}$ and $\phi_{\mathrm{out}}^{(m)}(R+\ell) = \phi_{\mathrm{shell}}^{(m)}$. For simplicity, we linearize the reaction flux, $s(\phi_{\mathrm{out}}^{(m)}) \approx \Gamma_{\mathrm{out}} - k_{\mathrm{out}}\,\phi_{\mathrm{out}}$, where $\Gamma_{\mathrm{out}} = [\phi_{\mathrm{shell}}^{(m)}\,s(\phi_{\mathrm{out}}^{\mathrm{eq}}) - \phi_{\mathrm{out}}^{\mathrm{eq}}\,s(\phi_{\mathrm{shell}}^{(m)})]/(\phi_{\mathrm{shell}}^{(m)} - \phi_{\mathrm{out}}^{\mathrm{eq}})$ and $k_{\mathrm{out}} = [s(\phi_{\mathrm{out}}^{\mathrm{eq}}) - s(\phi_{\mathrm{shell}}^{(m)})]/(\phi_{\mathrm{shell}}^{(m)} - \phi_{\mathrm{out}}^{\mathrm{eq}})$. Using the diffusivity D_{out}, we can then determine the local fluxes at the droplet surface in the normal direction, $\boldsymbol{j}_{\mathrm{out}}^{(m)}\cdot\boldsymbol{n} = -D_{\mathrm{out}}\partial_r\phi_{\mathrm{out}}^{(m)}|_{r=R}$, which in 1, 2, and 3 dimensions read

$$\boldsymbol{j}_{\mathrm{out,1D}}^{(m)}\cdot\boldsymbol{n} = D_{\mathrm{out}}\frac{(\phi_{\mathrm{out}}^{\mathrm{eq}}\,k_{\mathrm{out}} - \Gamma_{\mathrm{out}})\cosh\left(\frac{\ell}{\xi_{\mathrm{out}}}\right) - \left(\phi_{\mathrm{shell}}^{(m)}\,k_{\mathrm{out}} - \Gamma_{\mathrm{out}}\right)}{k_{\mathrm{out}}\,\xi_{\mathrm{out}}\,\sinh\left(\frac{\ell}{\xi_{\mathrm{out}}}\right)} \tag{2a}$$

$$\boldsymbol{j}_{\mathrm{out,2D}}^{(m)}\cdot\boldsymbol{n} = D_{\mathrm{out}}\frac{(\phi_{\mathrm{out}}^{\mathrm{eq}}\,k_{\mathrm{out}} - \Gamma_{\mathrm{out}})\left[I_1\left(\frac{R}{\xi_{\mathrm{out}}}\right)K_0\left(\frac{\ell+R}{\xi_{\mathrm{out}}}\right) + K_1\left(\frac{R}{\xi_{\mathrm{out}}}\right)I_0\left(\frac{\ell+R}{\xi_{\mathrm{out}}}\right)\right] - \frac{\xi_{\mathrm{out}}}{R}\left(\phi_{\mathrm{shell}}^{(m)}\,k_{\mathrm{out}} - \Gamma_{\mathrm{out}}\right)}{k_{\mathrm{out}}\,\xi_{\mathrm{out}}\left[K_0\left(\frac{R}{\xi_{\mathrm{out}}}\right)I_0\left(\frac{\ell+R}{\xi_{\mathrm{out}}}\right) - I_0\left(\frac{R}{\xi_{\mathrm{out}}}\right)K_0\left(\frac{\ell+R}{\xi_{\mathrm{out}}}\right)\right]} \tag{2b}$$

$$\boldsymbol{j}_{\mathrm{out,3D}}^{(m)}\cdot\boldsymbol{n} = D_{\mathrm{out}}\frac{(\phi_{\mathrm{out}}^{\mathrm{eq}}\,k_{\mathrm{out}} - \Gamma_{\mathrm{out}})\left(R\coth\left(\frac{\ell}{\xi_{\mathrm{out}}}\right) + \xi_{\mathrm{out}}\right) - \frac{\ell+R}{\sinh\left(\frac{\ell}{\xi_{\mathrm{out}}}\right)}\left(\phi_{\mathrm{shell}}^{(m)}\,k_{\mathrm{out}} - \Gamma_{\mathrm{out}}\right)}{k_{\mathrm{out}}\,\xi_{\mathrm{out}}\,R}, \tag{2c}$$

where we introduced the reaction-diffusion length scale $\xi_{\mathrm{out}} = \sqrt{D_{\mathrm{out}}/|k_{\mathrm{out}}|}$, and I_α and K_α are the modified Bessel functions of the first and second kind, respectively. Note that the definitions for Γ_{out}, k_{out} diverge when $\phi_{\mathrm{shell}}^{(m)} \approx \phi_{\mathrm{out}}^{\mathrm{eq}}$. We then simply approximate the reaction flux, $s(\phi_{\mathrm{out}}^{(m)}) \approx \overline{\Gamma}_{\mathrm{out}}$, where $\overline{\Gamma}_{\mathrm{out}} = [s(\phi_{\mathrm{shell}}^{(m)}) + s(\phi_{\mathrm{out}}^{\mathrm{eq}})]/2$. As before, we solve Eq. (7) in the main text in stationary state assuming spherical symmetry along with the boundary conditions $\phi_{\mathrm{out}}^{(m)}(R) = \phi_{\mathrm{out}}^{\mathrm{eq}}$ and $\phi_{\mathrm{out}}^{(m)}(R+\ell) = \phi_{\mathrm{shell}}^{(m)}$. After obtaining $\phi_{\mathrm{out}}^{(m)}$ inside the shell, the fluxes $\boldsymbol{j}_{\mathrm{out}}^{(m)}\cdot\boldsymbol{n}$ in 1, 2 and 3 dimensions then read as

$$\boldsymbol{j}_{\mathrm{out,1D}}^{(m)}\cdot\boldsymbol{n} = \frac{D_{\mathrm{out}}\,(\phi_{\mathrm{out}}^{\mathrm{eq}} - \phi_{\mathrm{shell}}^{(m)})}{\ell} - \frac{\overline{\Gamma}_{\mathrm{out}}\,\ell}{2} \tag{3a}$$

$$\boldsymbol{j}_{\mathrm{out,2D}}^{(m)}\cdot\boldsymbol{n} = \frac{\overline{\Gamma}_{\mathrm{out}}\,\ell\,(\ell+2R) - 4\,\phi_{\mathrm{out}}^{\mathrm{eq}}\,D_{\mathrm{out}} + 4\,\phi_{\mathrm{shell}}^{(m)}\,D_{\mathrm{out}}}{4\,R\,\log\left(\frac{R}{\ell+R}\right)} + \frac{\overline{\Gamma}_{\mathrm{out}}\,R}{2} \tag{3b}$$

$$\boldsymbol{j}_{\mathrm{out,3D}}^{(m)}\cdot\boldsymbol{n} = -\frac{\overline{\Gamma}_{\mathrm{out}}\,\ell^2\,(\ell+3R) - 6\,\phi_{\mathrm{out}}^{\mathrm{eq}}\,D_{\mathrm{out}}(\ell+R) + 6\,\phi_{\mathrm{shell}}^{(m)}\,D_{\mathrm{out}}(\ell+R)}{6\,\ell\,R}. \tag{3c}$$
